# From Divergent Pathogenesis to Precision Medicine: The Road to Targeted Therapy in HIV-Associated and HIV-Unrelated Diffuse Large B-Cell Lymphoma

**DOI:** 10.3390/diagnostics16193242

**Published:** 2026-10-08

**Authors:** Stephanie Murugan, Zivanai Cuthbert Chapanduka, Nadine Rapiti

**Affiliations:** 1 School of Laboratory Medicine and Health Sciences, University of KwaZulu Natal, Private Bag 7, Congella, Durban 4013, South Africa; rapitin@ukzn.ac.za; 2 National Health Laboratory Service (NHLS), Department of Haematology, KwaZulu-Natal Academic Complex, Inkosi Albert Luthuli Hospital, Private Bag X03, Mayville, Durban 4058, South Africa; 3 School of Laboratory Medicine and Health Sciences, University of Stellenbosch, Private Bag X1, Stellenbosch 7599, South Africa; zivanai@sun.ac.za

**Keywords:** diffuse large B-cell lymphoma, human immunodeficiency virus (HIV), pathogenesis, molecular biology

## Abstract

Diffuse large B-cell lymphoma (DLBCL) is the most common and biologically heterogeneous subtype of non-Hodgkin lymphoma. While immunochemotherapy has improved outcomes, survival remains variable due to substantial molecular diversity. In people living with HIV (PLWH), DLBCL is the predominant lymphoma subtype and demonstrates more aggressive clinical behaviour, characterised by advanced-stage disease, extranodal involvement, and a potentially poorer prognosis despite antiretroviral therapy. Clarifying differences in the pathogenesis of HIV-associated and HIV-unrelated DLBCL is essential for informing precision therapies. This narrative review highlights the contrasting pathogenic mechanisms, with an emphasis on the interplay between viral oncogenesis, host immunity, and molecular alterations in HIV-associated DLBCL versus DLBCL in immunocompetent individuals. Understanding these differences is critical for refining diagnostic approaches, improving risk stratification, and identifying novel therapeutic strategies tailored to both groups.

## 1. Introduction

Diffuse large B-cell lymphoma (DLBCL) is the most common and molecularly heterogeneous subtype of non-Hodgkin lymphoma (NHL), comprising approximately 30% of all NHL cases in developed countries and an even higher proportion in developing countries, with approximately 60% reported in some African countries [1,2]. Despite improvements in immunochemotherapy, outcomes remain variable, largely due to the profound genetic and phenotypic diversity that characterises this entity [3,4]. Increasing evidence suggests that therapeutic response is determined not only by clinical factors but also by the underlying molecular drivers of the disease, making precision medicine a central focus of contemporary DLBCL management.

The challenge of biological heterogeneity is particularly relevant in people living with human immunodeficiency virus (HIV). HIV is one of the world’s greatest health challenges, with approximately 39 million people living with HIV infection (PLWH) globally, of whom an estimated 25.6 million are in Africa [5]. With the advent of antiretroviral therapy (ART), the life expectancy of PLWH has increased, resulting in malignancies becoming an important cause of morbidity and mortality [6]. DLBCL is the most common NHL subtype affecting PLWH, comprising about 50% of all lymphomas seen in this group [7]. Despite improvements in HIV care, clinical outcomes remain variable, suggesting that conventional treatment approaches do not fully account for the unique biological context in which these tumours develop [8,9,10].

Although HIV-associated and HIV-unrelated DLBCL share many morphological and immunophenotypic features, accumulating evidence indicates that they arise through partially distinct pathogenetic mechanisms. The pathogenesis of HIV-associated DLBCL reflects a complex interplay between chronic antigenic stimulation, immune dysregulation, and oncogenic viral co-infections. Chronic immune activation and impaired immune surveillance promote B-cell hyperproliferation, somatic hypermutation, and genomic instability, thus fostering lymphomagenesis [11,12]. Conversely, in immunocompetent hosts, DLBCL more frequently harbours cell-intrinsic oncogenic alterations that drive the emergence of distinct molecular subtypes, each characterised by unique pathogenetic mechanisms. These processes converge on many of the same oncogenic pathways but may generate distinct molecular dependencies that influence disease behaviour and therapeutic susceptibility.

This narrative review focuses on comparing the pathogenesis and molecular biology of DLBCL in HIV-negative and HIV-positive patients. By mapping how distinct pathogenetic mechanisms define actionable vulnerabilities, this review aims to highlight the translational relevance of divergent biology for advancing precision therapy in DLBCL.

## 2. Methodology

This narrative review was conducted using a structured literature search designed to comprehensively identify published evidence relating to the pathogenesis, molecular biology, tumour microenvironment, and targeted therapeutic strategies in HIV-associated and HIV-unrelated DLBCL. Although this review was not conducted as a formal systematic review, the search strategy was designed to ensure transparency, reproducibility, and broad coverage of the available literature.

Searches were performed across two major databases (PubMed and Semantic Scholar) covering the last 10 years (2015–2025). Search terms were developed using combinations of Medical Subject Headings (MeSH) and free-text keywords, including: “diffuse large B-cell lymphoma,” “DLBCL,” “HIV-associated DLBCL,” “HIV-related lymphoma,” “lymphomagenesis,” “molecular pathogenesis,” “genomics,” “epigenetics,” “tumour microenvironment,” “precision medicine,” “targeted therapy,” “B-cell receptor signalling,” “NF-κB,” “PI3K/AKT/mTOR,” and “Epstein–Barr virus.” Boolean operators (“AND” and “OR”) were used to refine the search and identify relevant studies.

All retrieved records (*n* = 1910) were imported into a reference manager. Case studies, duplicate entries, conference abstracts, editorials, and non-peer-reviewed literature (*n* = 1042) were removed.

Studies were considered eligible if they investigated the molecular mechanisms, genetic alterations, immune dysregulation, tumour microenvironment, or therapeutic targeting of HIV-associated or HIV-unrelated DLBCL. Original research articles, clinical trials, translational studies, systematic reviews, meta-analyses, and high-quality narrative reviews were included. Studies were excluded if they focused exclusively on other lymphoma subtypes without relevance to DLBCL, were published in languages other than English, were conference abstracts lacking sufficient methodological detail, or were editorials, opinion articles, or duplicate reports. Where multiple publications described overlapping patient cohorts, the most comprehensive or recent study was preferentially included.

For Tables 2–4, genetic alterations were selected from high-quality genomic, sequencing, and translational studies that consistently identified recurrent driver mutations, chromosomal rearrangements, and signalling pathway abnormalities in DLBCL. Preference was given to alterations that were recurrently reported across multiple studies and had recognised biological, prognostic, or therapeutic relevance.

A total of 868 manuscripts were screened at the title and abstract level by the primary author for relevance and duplication. This screening was followed by a full-text review of potentially eligible articles. Reference lists of selected publications were manually searched to identify additional relevant studies that were not captured during the electronic database search. The final selection of articles (*n* = 219) was based on their scientific quality, relevance to the objectives of the review, and contribution to the current understanding of DLBCL biology and precision medicine.

The evidence presented in this review was synthesised narratively, with emphasis on comparing the biological mechanisms underlying HIV-associated and HIV-unrelated DLBCL and critically evaluating their implications for precision medicine. Particular attention was given to areas of agreement and controversy within the literature, emerging therapeutic targets, and existing knowledge gaps requiring further investigation.

As a narrative review, this study has inherent limitations. Formal assessment of study quality or risk of bias was not performed, and publication bias cannot be excluded. Nevertheless, the inclusion of multiple databases, recent genomic and translational studies, and manual reference screening was intended to provide a comprehensive and balanced overview of the current evidence.

## 3. Molecular Pathogenesis of DLBCL

DLBCL is a genetically diverse lymphoma driven by specific patterns of genetic alterations that disrupt normal B-cell development. Most DLBCL cases have no known causative factor and arise de novo. A small proportion (3–10%) can occur as a result of transformation from an indolent B-cell lymphoma, reflecting the multistep evolutionary nature of lymphomagenesis [13]. Rather than being driven by a single oncogenic lesion, DLBCL develops through the cumulative acquisition of molecular abnormalities that disrupt normal germinal centre (GC) biology, deregulate cell survival pathways, impair differentiation, and enable immune evasion.

Over the past two decades, genomic and transcriptomic profiling has transformed the understanding of DLBCL biology. Although the traditional cell-of-origin (COO) classification distinguishes germinal centre B-cell (GCB) and activated B-cell (ABC) subtypes, it does not fully capture the disease’s molecular heterogeneity. Recent genomic studies have demonstrated that DLBCL comprises multiple genetically defined subgroups with distinct pathogenic mechanisms, prognostic outcomes, and therapeutic vulnerabilities [14,15,16,17]. Understanding the molecular events that drive lymphomagenesis is no longer solely of biological interest but has become essential for the development of precision medicine strategies.

### 3.1. Disrupted Germinal Center Biology

The pathogenesis of DLBCL reflects a convergence of molecular and cellular disruptions that frequently arise during the GC reaction of B cells. The GC, a specialised microenvironment within secondary lymphoid organs, serves as the site of B-cell maturation, clonal expansion, and affinity-driven selection. These processes are tightly regulated under physiological conditions to ensure effective antibody responses [18,19]. Although these processes are essential for adaptive immunity, they also create an intrinsically mutagenic environment in which genomic instability is an unavoidable consequence of normal B-cell maturation.

Under physiological conditions, GC reactions are tightly regulated through coordinated interactions between B cells, follicular dendritic cells, T-follicular helper cells, and stromal cells. These interactions ensure that only B cells expressing high-affinity antigen receptors survive, while cells acquiring deleterious mutations undergo apoptosis. DLBCL develops when this quality-control process fails, allowing genetically altered B cells to evade apoptosis and continue proliferating.

A mature GC is compartmentalised into the dark zone (DZ) and light zone (LZ), each contributing to distinct stages of B-cell development. The DZ consists of rapidly proliferating centroblasts, characterised by low surface immunoglobulin expression, absence of B-cell lymphoma 2 (BCL2), and intrinsic apoptotic susceptibility. Here, B cells undergo somatic hypermutation (SHM), generating a highly diverse immunoglobulin repertoire. Aberrations in SHM or its regulatory machinery can introduce off-target mutations into proto-oncogenes and tumour suppressor genes, thereby initiating lymphomagenesis [20].

In the LZ, centrocytes bearing high-affinity surface immunoglobulins compete for survival signals from follicular dendritic cells, T follicular helper cells, and tingible body macrophages. Under normal conditions, this ensures the positive selection of B cells with superior antigen affinity. However, dysregulation of these survival pathways, such as constitutive activation of B-cell receptor (BCR) signalling, aberrant nuclear factor kappa-light-chain-enhancer of activated B cells (NF-κB) pathway activation, or impaired apoptosis due to BCL2 overexpression, facilitates the persistence of defective clones [19].

Following positive selection, B cells may undergo additional cycles of proliferation in the DZ or proceed through class-switch recombination (CSR) to acquire specialised effector functions. Dysregulated CSR, mediated by aberrant activity of activation-induced cytidine deaminase (AID), may contribute to chromosomal translocations involving the oncogenes B-cell lymphoma 6 (BCL6) (3q27), myelocytomatosis oncogene (MYC) (8q24) and BCL2 (18q21), which are characteristic of DLBCL pathogenesis [21,22]. Ultimately, instead of differentiating into long-lived plasma cells or memory B cells, these genetically altered clones can accumulate, driving malignant transformation.

**Figure 1:**
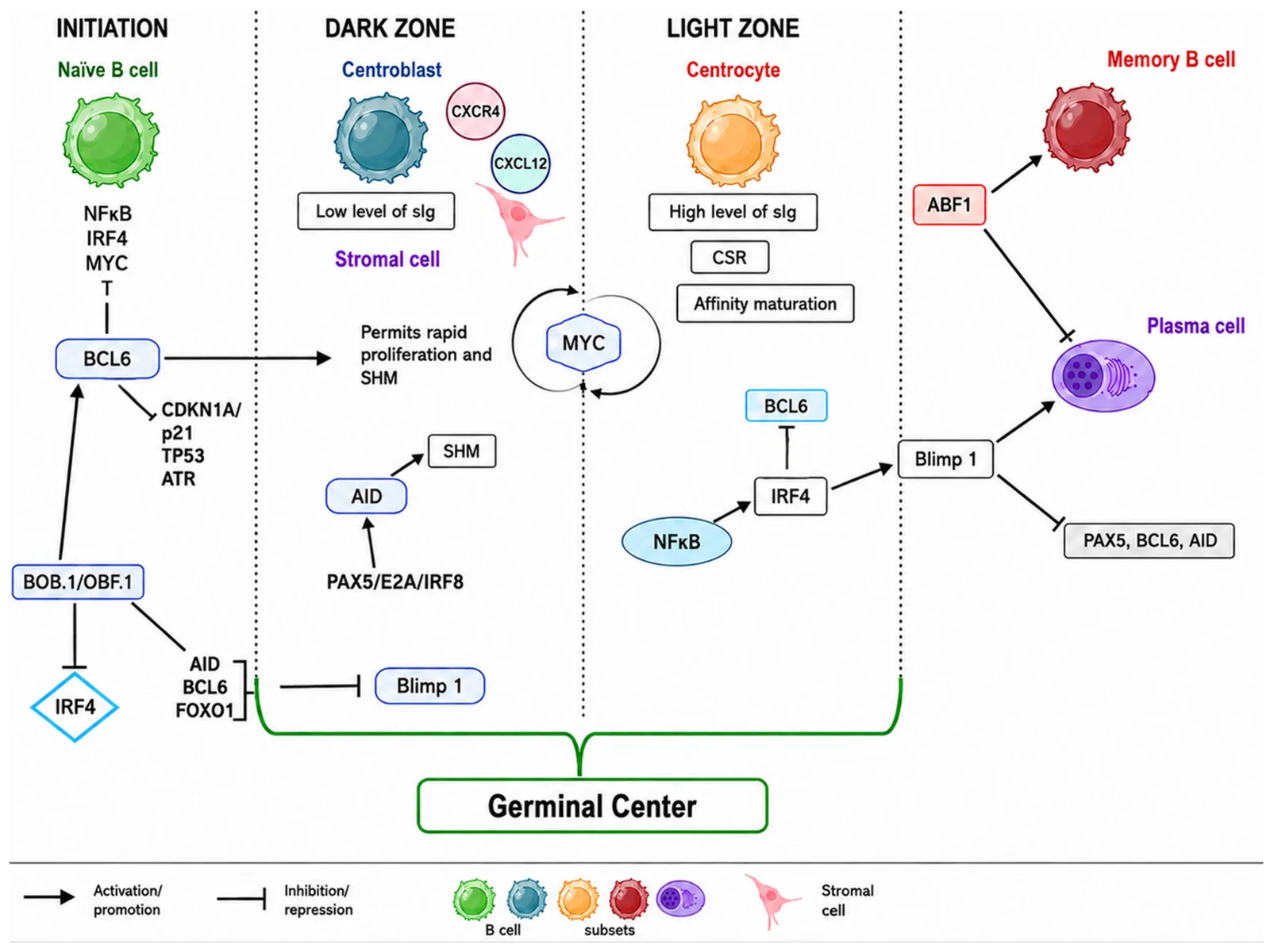
Transcription Factors (TFs) regulating Germinal Centre (GC) biology: B-cell lymphoma 6 (BCL6) serves as a master regulator, promoting rapid proliferation, somatic hypermutation (SHM) and GC migration by suppressing cell cycle inhibitors (CDKN1A/p21) and DNA damage checkpoints (TP53, ATR). Activation -induced cytidine deaminase (AID), crucial for SHM, is positively regulated by PAX5, EZH2, and IRF8. In the GC dark zone (DZ), high FOXO1 expression cooperates with BCL6 to repress the plasma cell differentiation factor BLIMP1, while BCL6 suppresses the memory B cell driver ARS-binding factor 1 (ABF1). Conversely, in the light zone (LZ) IRF4 upregulation downregulates BCL6 and induces BLIMP1 to drive plasma cell fate. A subset of LZ B cells re-express MYC to govern DZ re-entry for clonal selection. Oct-binding factor 1 (OBF1/BOB.1) orchestrates the entire program by binding to and activating BCL6 and AICDA while repressing IRF4.

Several transcription factors (TFs) play a role in the initiation and maintenance of the GC (Figure 1). Dysregulation of these TFs contributes significantly to tumourigenesis in DLBCL [23].

### 3.2. Molecular Classification of DLBCL

Gene expression profiling (GEP) studies introduced in the 2000s broadly divided DLBCL into two main molecular subtypes, GCB and ABC, which are morphologically indistinguishable on histology. A proportion of cases (10–15%) cannot be classified into either profile [24]. The distinct genes expressed in each subgroup reflect the different stages of B-cell development from which the malignant B-cell arises and the possible COO.

GCB DLBCL originates from GC centroblasts and express genes similar to those expressed by normal GC B-cells, including CD10 and LIM-only protein 2 (LMO2), with high expression of BCL 6.

ABC DLBCL originates from post-GC B-cells blocked from plasmacytic differentiation. This subtype expresses several genes that are normally upregulated in plasma cells, together with activated chronic BCR signalling. These include NF-κB and interferon regulatory factor 4 (IRF4)/multiple myeloma oncogene-1 (MUM1). These cells are blocked from full differentiation into plasma cells by additional genetic alterations. These tumours are enriched for mutations affecting the BCR pathway.

Although the COO classification has provided important biological insight into DLBCL, it has significant limitations. It does not adequately capture the genetic heterogeneity within the GCB and ABC subtypes, lacks methodological standardization, and has limited influence on frontline treatment decisions.

The acknowledged limitations of the COO model have precipitated a paradigm shift toward next-generation sequencing (NGS)-based genomic classifications (Table 1). Modern frameworks, such as the LymphGen algorithm, have redefined the DLBCL landscape by clustering tumours according to their unique co-mutational and structural alteration profiles. This genomic resolution is clinically vital, as specific high-risk molecular drivers frequently override traditional COO boundaries. These genetically defined subtypes (MCD, BN2, N1, EZB, ST2, and AT3) are also now recognised by both the International Consensus Classification (ICC) and World Health Organisation (WHO), as they better predict prognosis and identify potential therapeutic targets [25,26].

### 3.3. Genetic Alterations in DLBCL

Mechanisms of genetic alteration in DLBCL involve chromosomal translocations, mutations caused by aberrant somatic hypermutation (ASHM), sporadic somatic mutations, and copy number alterations. Chromosomal translocations in DLBCL rarely generate fusion proteins. Instead, they typically reposition heterologous regulatory elements, such as promoters or enhancers from partner chromosomes, adjacent to intact proto-oncogene coding regions. This process most frequently involves immunoglobulin (Ig) loci and leads to aberrant overexpression of otherwise physiologically normal proteins.

SHM is a physiological mechanism confined to GC B cells, in which AID introduces point mutations into immunoglobulin variable (IgV) region genes to optimise antigen affinity. Under normal conditions, SHM is stringently regulated and spatially restricted to Ig loci. In DLBCL, however, this process becomes dysregulated, extending beyond its canonical targets in a phenomenon known as ASHM. ASHM is defined by the off-target activity of AID on non-Ig genes, particularly proto-oncogenes such as BCL6, MYC, Paired Box Gene 5 (PAX5), and PIM1 [20]. These mutations occur predominantly within promoter and regulatory regions, driving oncogene activation and contributing to malignant transformation. Given its role in lymphoma initiation and progression, ASHM has emerged as a potential therapeutic vulnerability. Strategies aimed at inhibiting AID activity or modulating downstream DNA repair pathways are being explored as approaches to reduce the mutational burden in B-cell lymphomas.

Multiple genetic lesions have been described in GCB DLBCL and ABC DLBCL, with some alterations common to both subtypes (Table 2, Table 3 and Table 4).

## 4. Pathogenesis of HIV-Associated DLBCL 

HIV belongs to the *Lentivirinae* subfamily of retroviruses, a group of ribonucleic acid (RNA) viruses capable of establishing chronic cellular infection through reverse transcription of their RNA genome into a deoxyribonucleic acid (DNA) provirus, which subsequently integrates into the host genome. Two distinct types of HIV have been identified, HIV-1 and HIV-2, which are distinguished by genetic variation and antigenic properties. The pathophysiology of HIV infection is characterised by progressive immune system deterioration, ultimately culminating in the development of AIDS.

The aetiopathogenesis of HIV-associated DLBCL involves a complex interplay of direct and indirect factors stemming from chronic HIV infection. It is a multistep process driven by chronic immune deregulation, chronic inflammation, co-infection with oncogenic viruses, and indirect effects of HIV [7,11].

### 4.1. Immunodeficiency and Chronic Inflammation

Severe immunosuppression in HIV results from profound T and B cell dysregulation, which leads to defective immune surveillance. Various mechanisms are responsible for the immune dysfunction associated with HIV infection. Continuous immune activation and inflammation alter thymic function, leading to inefficient thymopoiesis, atrophy, and fibrosis, with resultant exhaustion of T cells and aberrant development of regulatory T cells (Tregs). This creates an environment conducive to opportunistic infections and inadequate HIV control [11].

**Figure 2:**
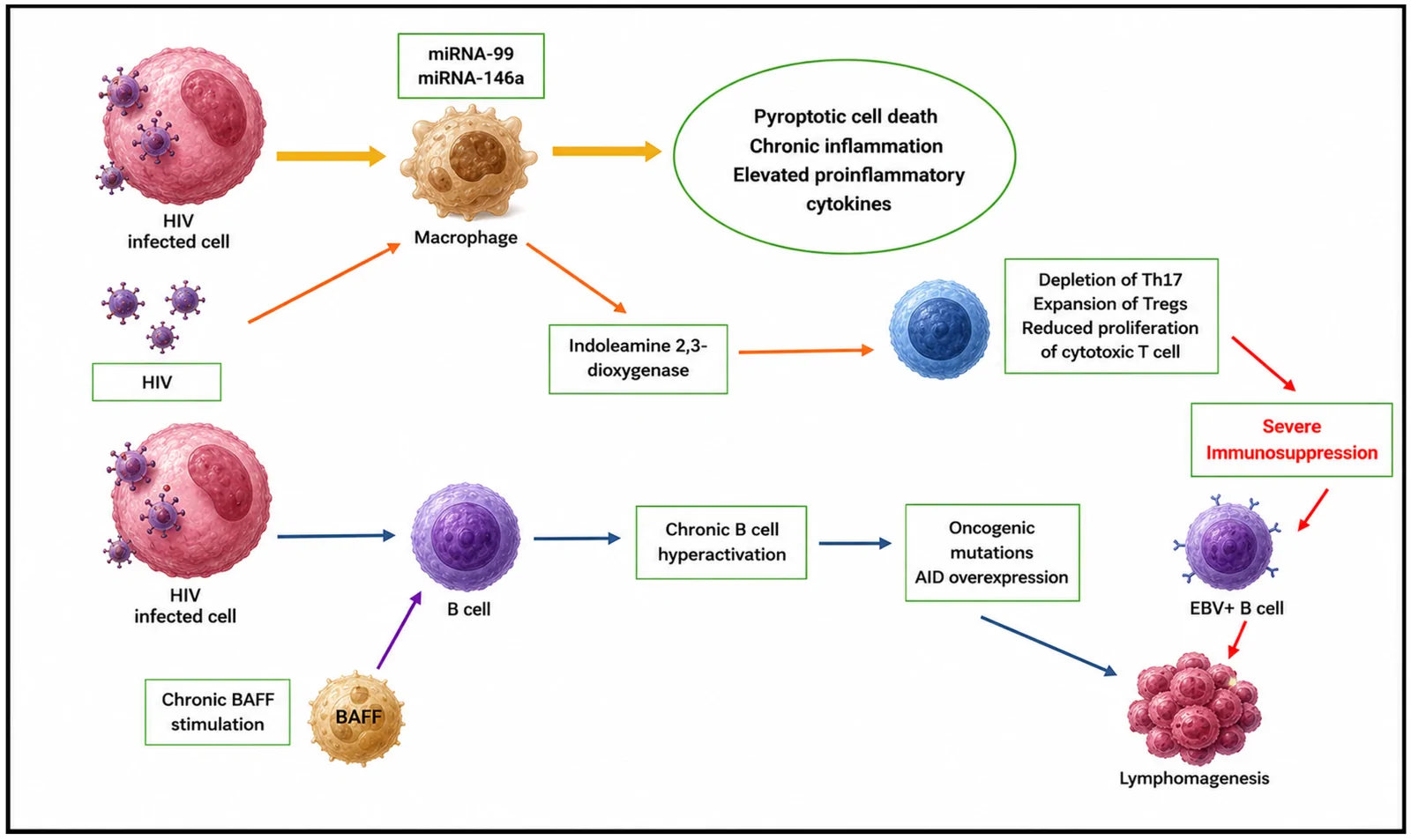
Immunodeficiency and chronic inflammation during HIV infection. Schematic illustrating how chronic inflammation and immunodeficiency promote lymphomagenesis in people living with HIV. BAFF—B-cell-activating factor, AID—activation-induced cytidine deaminase.

HIV-infected macrophages demonstrate upregulated expression of microRNA (miRNA)-99 and miRNA-146a (Figure 2). miRNA-99 impairs intracellular pathways responsible for viral degradation, thereby facilitating the persistence of HIV within these cells. The sustained presence of infected macrophages contributes to pyroptotic cell death and perpetuates chronic inflammation, marked by elevated secretion of proinflammatory cytokines, including tumour necrosis factor-alpha (TNFα), interleukin-6 (IL-6), and interleukin-1β (IL-1β). Concurrently, miRNA-146a represses innate immune signaling through downregulation of C-C motif chemokine ligand 5 (CCL5), resulting in reduction in immune cell recruitment and persistence of viral infection [49].

The HIV proteins Tat and Nef enhance the production of the indoleamine 2,3-dioxygenase enzyme by macrophages and dendritic cells [11,50]. This leads to depletion of T helper 17 (Th17) cells, expansion of Treg cells, and reduced proliferation of cytotoxic T cells. Natural killer (NK) cells are also affected by HIV infection. HIV reduces the expression of NK receptor group 2 member D (NKG2D) and natural cytotoxicity receptors (NCRs). This results in an impaired ability of NK cells to recognise precancerous tissue, increasing cancer risk [51].

HIV infection is associated with chronic B-cell hyperactivation, driven by persistent antigenic stimulation from the virus itself, co-infecting pathogens, and microbial translocation from the gastrointestinal tract. Elevated concentrations of B-cell activating cytokines, such as IL-6 and IL-10, foster an environment of uncontrolled B-cell proliferation and increased genomic instability. This state of sustained activation enhances the likelihood of acquiring oncogenic mutations, most commonly involving BCL2, BCL6, tumour protein p53 (TP53), and MYC, thereby predisposing cells to malignant transformation.

An additional mechanism of aberrant B-cell activation involves the CD40 ligand (CD40L), a co-stimulatory molecule normally expressed on activated T cells that promotes B-cell maturation and high-affinity antibody production through binding to CD40 on B cells. In PLWH, CD40L is incorporated into virions, producing CD40L-bearing HIV particles capable of engaging CD40 on B cells in a dysregulated manner, thereby mimicking and amplifying physiological stimulation [11,52].

B-cell hyperactivation also dysregulates the expression of AID. AID overexpression introduces point mutations, deletions, substitutions, and duplications in proto-oncogenes, including BCL6, MYC, and PIM1, while also driving chromosomal translocations that juxtapose BCL2, BCL6, or MYC with immunoglobulin heavy-chain loci on chromosome 14, resulting in their inappropriate overexpression. Furthermore, AID is upregulated through interactions between the HIV envelope glycoprotein gp120 and C-type lectin receptors on B cells [53].

B-cell–activating factor (BAFF), a critical regulator of B-cell survival, differentiation, and antibody production, is elevated in PLWH [54]. Chronic BAFF stimulation suppresses NR4A transcription factors, disrupting B-cell tolerance, impairing immune regulation, and contributing to B-cell exhaustion (Figure 2).

Immunosuppression and co-infection with viruses encoding oncogenic proteins contribute to lymphoma formation in HIV-positive patients. Epstein–Barr virus (EBV) occurs in 90% of DLBCL with immunoblastic morphology and 30% of DLBCL with centroblastic morphology [55]. EBV is a gamma-herpes virus with the ability to infect B lymphocytes through interactions with CD21. Chronic B-cell activation and infection with EBV upregulate cellular receptors such as c-x-c motif chemokine receptor 2 (CXCR2) for HIV matrix protein p17 variants. CXCR2 upregulation by different p17 variants promotes B-cell expansion, increasing the probability of acquiring genetic alterations. p17 variants in EBV-infected B-cells may also upregulate latent membrane protein 1 (LMP-1), which is the main EBV carcinogenic protein. The effect of LMP-1 on B lymphocytes mimics that of physiological CD40 signalling, stimulating lymphocyte proliferation and antiapoptotic pathways in an NFκB- and Akt-dependent manner. EBV-encoded nuclear antigen 2 (EBNA2) also plays a role in lymphomagenesis by exhibiting pro-survival activity.

BLIMP1 is highly expressed in EBV-positive HIV-associated DLBCL, giving these cells the ability to evade apoptosis. In addition, CD30 expression is associated with HIV-associated DLBCL with EBV infection. CD30 stimulates activation of the NFkB pathway, which is associated with cellular proliferation and carcinogenesis [55].

### 4.2. Direct Mechanisms: HIV Viral Proteins

**Figure 3:**
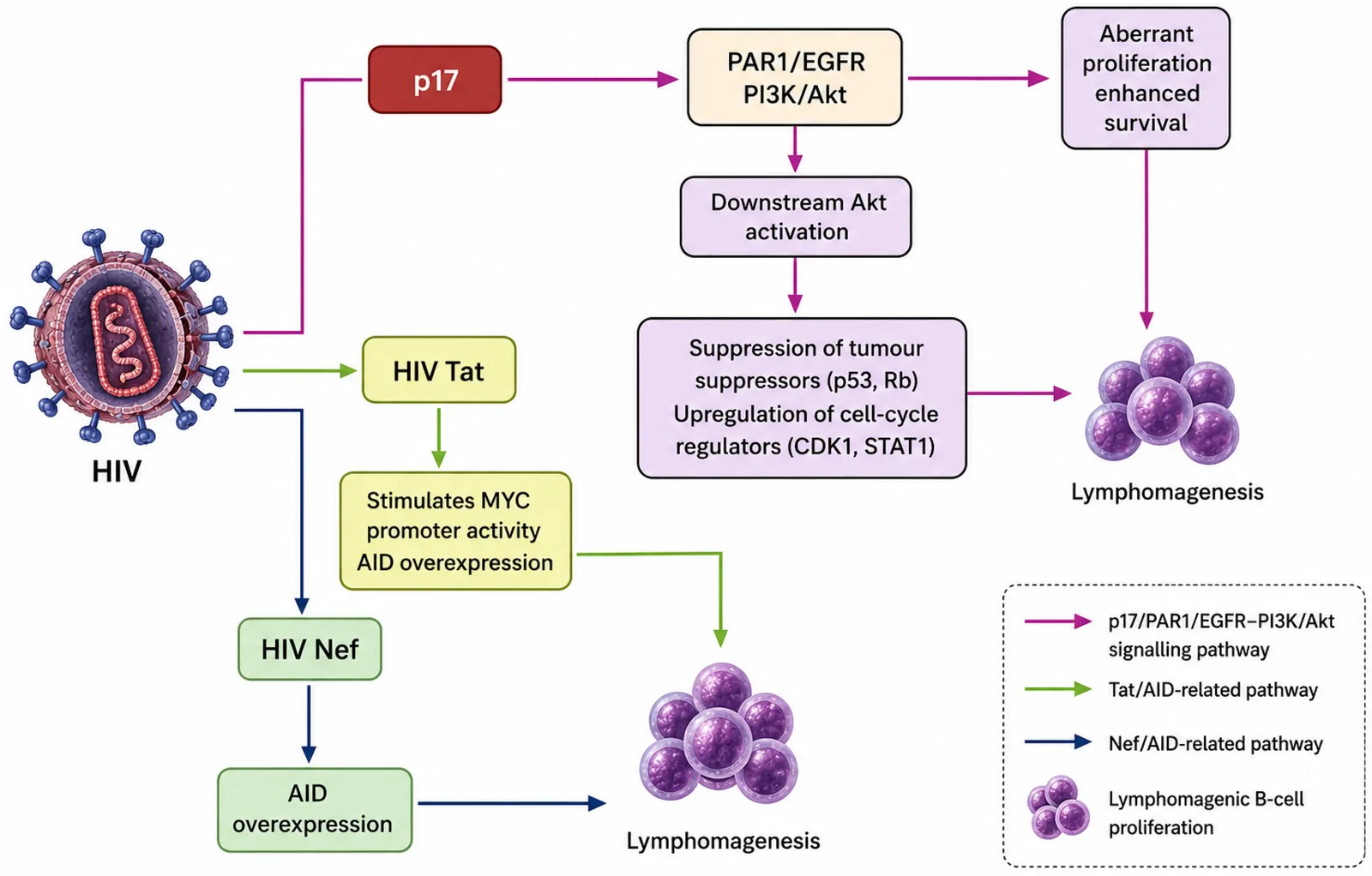
HIV viral proteins and lymphomagenesis. HIV viral proteins and immune dysfunction create an environment that favours B-cell activation, prolonged survival, and genomic instability.

HIV viral proteins, together with the accompanying immune dysfunction, create a microenvironment that favours B-cell activation, prolonged survival, and genomic instability, which are key drivers of malignant transformation (Figure 3).

The HIV matrix protein p17, a 132–amino acid structural protein essential for viral assembly and release, exerts direct oncogenic effects. p17 engages protease-activated receptor 1 (PAR1), initiating the PAR1/EGFR/PI3K/Akt signalling cascade, which promotes aberrant proliferation, enhanced survival, and lymphoma development [11,56]. Downstream Akt activation regulates multiple cell-cycle and survival mediators, including cyclin-dependent kinase 1 (CDK1), retinoblastoma protein (Rb), signal transducer activator of transcription factor 1 (STAT1), protein tyrosine phosphatase 1B (PTP1B), and tumour suppressor protein (p53). Through suppression of tumour suppressors (p53, Rb) and upregulation of cell-cycle regulators (CDK1, STAT1), Akt signalling provides a potent survival advantage to transformed B cells. Furthermore, a structural variant of p17, containing the AT20 loop, binds c-x-c motif chemokine receptors (CXCR1, CXCR2), activating G-protein–mediated signalling. This interaction drives endothelial proliferation and vascular remodelling via the PI3K/Akt and MEK/ERK1/2 pathways, implicating p17 in both angiogenic and lymphomagenic processes.

The HIV Tat protein also profoundly influences B-cell biology. Tat enhances transcription of the proto-oncogene MYC by binding to transcription factors such as activator protein 1 (AP-1) and Jun B, thereby stimulating MYC promoter activity [57,58]. B cells exposed to Tat exhibit increased chromosomal alterations and genomic instability. Tat directly enters B cells and promotes MYC–IgH rearrangements, leading to MYC overexpression. In addition, Tat activates the Akt/mTORC1 pathway and suppresses transcriptional repressors of activation-induced cytidine deaminase, thereby driving AID overexpression. These combined effects facilitate cell survival, mutagenesis, and malignant proliferation. The HIV Nef protein further contributes to lymphomagenesis by upregulating AID expression, thereby enhancing ASHM and genomic instability [59].

### 4.3. Tumour Microenvironment

The tumour microenvironment (TME), in conjunction with HIV-derived viral components, plays a pivotal role in driving lymphomagenesis in HIV-associated DLBCL [55,60]. Within the TME, tumour-associated macrophages (TAMs), NK cells, T cells, B cells, oncogenic viruses, and a network of cytokines collectively contribute to tumour initiation, progression, and immune evasion.

DLBCL in HIV-positive patients has markedly elevated angiogenic activity [55,60]. Neovascularisation is tumour-driven and linked to immunoblastic features and EBV infection. Angiogenesis is the physiological process through which new blood vessels are generated from pre-existing vasculature. In the context of cancer biology, it represents a critical mechanism that sustains tumour growth, facilitates nutrient and oxygen delivery, and enables metastatic dissemination. Angiogenic processes begin with endothelial cell activation, which is initiated by the transcription factor Hypoxia -Inducible Factor 1 (HIF 1) [61]. HIF1α induces proangiogenic and antiangiogenic factors such as vascular endothelial growth factor (VEGF), fibroblast growth factor (FGF), platelet-derived growth factor (PDGF), IL-6, and IL-8. Activation of receptors such as vascular endothelial growth factor receptor 2 (VEGFR2) stimulates several signalling pathways, which enhance angiogenesis by promoting cell proliferation, survival, and invasion.

The HIV Tat protein promotes angiogenesis through direct engagement and activation of VEGFR2 signalling pathways [62]. Notably, Tat can be secreted by HIV-infected cells, enabling it to function in a paracrine manner and exert effects independently of direct tumour cell infection. Likewise, the HIV matrix protein p17 displays IL-8–mimetic chemokine activity by binding to CXCR1 and CXCR2, thereby inducing endothelial cell proliferation and facilitating the formation of capillary-like vascular structures.

EBV also contributes to angiogenesis through LMP1-mediated signalling. LMP1 increases HIF1α by promoting its expression and downregulating its repressors [63].

### 4.4. Genomic and Transcriptomic Landscape of HIV-Associated DLBCL

Although HIV-associated and HIV-unrelated DLBCL share many recurrent driver alterations, accumulating genomic evidence suggests that the relative contribution of these alterations differs between the two diseases. Rather than representing a completely distinct genomic entity, HIV-associated DLBCL appears to evolve through a unique interplay between tumour-intrinsic genetic lesions, chronic immune activation, HIV-mediated immune dysregulation, and oncogenic viral co-infections. Defining the molecular landscape in HIV-associated DLBCL remains challenging because PLWH have been substantially underrepresented in large genomic sequencing studies and prospective clinical trials, with approximately 70% of lymphoma trials excluding this patient population [64]. Consequently, the molecular frameworks currently guiding precision medicine have been derived almost exclusively from HIV-negative cohorts, and their applicability to HIV-associated DLBCL remains uncertain.

Comparative genomic studies demonstrate that several hallmark driver alterations are shared between HIV-associated and HIV-unrelated DLBCL. Baptista et al. demonstrated that the frequency of MYC and BCL6 rearrangements in HIV-associated DLBCL patients was similar to that found in immunocompetent patients with DLBCL [65]. This suggests that these oncogenic events remain fundamental regardless of HIV status. In contrast, BCL2 rearrangements were consistently less frequent in HIV-associated DLBCL than in immunocompetent patients, a finding confirmed across multiple studies [65,66]. This relative lack of BCL2 abnormalities suggests that HIV-associated lymphomagenesis may depend less on classical anti-apoptotic escape mechanisms and more on chronic immune activation, inflammatory signalling, and virus-driven oncogenesis.

Frequent alterations involving TP53, together with ASHM involving PIM1 and PAX5, have also been reported in ~50% of cases, further supporting the contribution of genomic instability to tumour development [55,67].

Although available genomic studies remain relatively small, they suggest that HIV-associated DLBCL may possess a broader and more heterogeneous mutational spectrum than HIV-unrelated disease. In a Chinese cohort of twelve HIV-positive patients, Yu Peng et al. identified a higher frequency of mutations involving low-density lipoprotein receptor-related protein 1 (LRP1), MYC, and tyrosine kinase (TYK) compared with HIV-negative controls [68]. While these findings require validation in larger cohorts, they support the hypothesis that HIV infection influences both the diversity and selection of oncogenic mutations.

One of the most striking biological differences between HIV-associated and HIV-unrelated DLBCL is the influence of EBV on the genomic landscape. EBV-positive HIV-associated DLBCL demonstrates recurrent alterations involving STAT3, DDX3X, and TP53, together with activation of the JAK/STAT and NF-κB signalling pathways [66,69]. Conversely, EBV-negative HIV-associated DLBCL more frequently harbours mutations affecting epigenetic regulators, including KMT2D and TET2 [66]. These observations indicate that HIV-associated DLBCL is itself biologically heterogeneous, comprising distinct molecular subgroups driven by different pathogenetic mechanisms. Such heterogeneity has important implications for precision medicine because therapeutic vulnerabilities are likely to differ according to both EBV status and the underlying molecular alterations.

Additional genomic studies have identified further differences between HIV-associated and HIV-unrelated DLBCL. A study conducted by Teitell et al. identified increased expression of the T-cell Leukemia (TCL) oncogene in AIDS-related DLBCL [70]. Subsequent studies demonstrated upregulation of microRNA-21, unique chromosomal alterations such as 3p14.3 deletions and 12q21.31 gains, and downregulation of tumour suppressor genes, including FHIT and WWOX [71,72,73]. In contrast, gains involving chromosome 18q appear to occur more frequently in HIV-unrelated DLBCL [72]. Collectively, these findings suggest that HIV infection shapes tumour evolution through mechanisms extending beyond conventional driver mutations alone.

Emerging transcriptomic and multi-omic studies further support the concept that HIV-associated DLBCL develops within a unique biological context. Compared with HIV-unrelated disease, HIV-associated DLBCL demonstrates greater dependence on inflammatory signalling, altered metabolic programming, epigenetic dysregulation, immune escape, and TME interactions. Rather than acting independently, these processes cooperate with tumour-intrinsic genomic alterations to influence disease behaviour and therapeutic response. Table 5 summarises the principal genomic, transcriptomic, and microenvironmental differences reported between HIV-associated and HIV-unrelated DLBCL.

Collectively, the available evidence indicates that HIV-associated DLBCL shares many canonical oncogenic pathways with HIV-unrelated disease, but differs in the relative importance of immune dysregulation, viral oncogenesis, epigenetic alterations, and tumour microenvironmental interactions. However, interpretation of these findings remains limited by small cohort sizes, heterogeneous sequencing platforms, geographical variation, and the persistent underrepresentation of PLWH in genomic studies and clinical trials. Consequently, it remains uncertain whether current molecular classification systems fully capture the biological diversity of HIV-associated DLBCL or accurately predict responses to emerging targeted therapies. Future precision medicine strategies should therefore integrate tumour genomics with viral status, immune profiling, and TME characteristics, rather than relying solely on tumour-intrinsic mutations, while dedicated genomic studies involving PLWH are urgently required to validate HIV-specific biomarkers and therapeutic targets.

## 5. Precision Medicine in HIV-Associated and HIV-Unrelated DLBCL

The development of precision medicine in DLBCL has been driven by advances in our understanding of the molecular mechanisms of lymphomagenesis, enabling targeted therapies directed at tumour-specific biological pathways. However, translating these advances into the management of HIV-associated DLBCL remains challenging because most genomic studies and clinical trials have predominantly included HIV-negative patients. Although HIV-associated and HIV-unrelated DLBCL share some molecular features, chronic immune activation, EBV co-infection, and a distinct TME may influence therapeutic response.

A comparative overview of the clinicopathological, molecular, and therapeutic features of HIV-associated and HIV-unrelated DLBCL is presented in Table 6, highlighting their similarities, key differences, and implications for precision medicine.

## 6. Translating Biological Vulnerabilities into Targeted Therapies in DLBCL

Combination chemo-immunotherapy with rituximab, cyclophosphamide, doxorubicin, vincristine and prednisone (R-CHOP) has remained the mainstay of first-line therapy for DLBCL for the past two decades. Only 60–70% of patients respond favourably to R-CHOP [78,79]. A primary obstacle to implementing effective targeted therapies in DLBCL lies in the extensive genetic and phenotypic heterogeneity of the disease.

Recent advances in genomic and molecular profiling of DLBCL have uncovered a spectrum of pathogenic drivers and signalling aberrations that extend far beyond the traditional histopathological classification. These insights have created a foundation for the development of novel targeted therapies aimed at specific oncogenic pathways, epigenetic lesions and microenvironmental interactions. By aligning therapeutic strategies with underlying biology of the disease, these agents hold promise not only for overcoming the limitations of conventional chemoimmunotherapy but also for improving outcomes in high-risk and refractory patient subsets.

### 6.1. Agents Targeting B-Cell Receptor (BCR) Signalling

The four BCR signalling targets of newer drug therapies include Bruton Tyrosine Kinase (BTK), Spleen Tyrosine Kinase (SYK), and Protein Kinase C Beta (PKCβ).

The non-receptor tyrosine kinase, BTK, plays a vital role in oncogenic signalling, essential for the proliferation and survival of leukemic cells in various B-cell malignancies. It acts as a key downstream kinase in the BCR signalling pathway [80]. The first-generation BTK inhibitor ibrutinib has shown promise in some DLBCL studies [81,82]. In a phase 2 clinical trial of 80 patients with relapsed DLBCL, ibrutinib monotherapy led to an overall response rate of 37% in patients with the ABC subtype, compared with only 5% of patients with the GCB subtype [83]. This differential response underscores the importance of molecular subtyping in guiding targeted therapies for DLBCL.

The phase III “Phoenix” trial (NCT01855750) demonstrated a survival advantage with the addition of ibrutinib to R-CHOP in younger patients with ABC DLBCL. Subsequent biopsy analyses from trial participants identified three defined genetic subtypes, MCD, BN2, and N1. Among younger patients (≤60 years), those with MCD or N1 subtypes achieved a 3-year event-free survival of 100% when treated with ibrutinib plus R-CHOP, whereas outcomes with R-CHOP alone were significantly worse (42.9% and 50%, respectively) [84].

Enzastaurin is a selective PKCβ inhibitor that inhibits signal transduction and ultimately pathway activation. The efficacy of this drug in DLBCL has not yet been proven. A multicentre phase 2 study demonstrated that, of the 55 patients enrolled in the study, 12 patients (22%; 95% CI, 13% to 46%) experienced freedom from progression (FFP) for two cycles, and eight patients remained free from progression for four cycles (15%; 95% CI, 6% to 27%). Four patients (7%; 95% CI, 2% to 18%), including three complete responders and one patient with stable disease, continued to experience FFP 20 to 50 months after study entry [85]. This drug may benefit patients with mutations in PKCβ, particularly when specific biomarkers are present.

SYK inhibitors are investigational drugs targeting SYK, a cytosolic non-receptor tyrosine kinase involved in B-cell signalling. SYK is hyperactivated in a significant portion of DLBCL cases, especially in the ABC subtype, making it a potential therapeutic target. However, studies have shown disappointing results. The results of an open-label phase II study of entospletinib (GS-9973) showed that none of the 43 patients with relapsed/refractory (R/R) DLBCL achieved a complete or partial response, and 26 patients (60%) had progressive disease [86]. Phase II trials using fostamatinib demonstrated that, despite the drug being well tolerated, its efficacy was limited. In this study, the overall response rate (ORR) was 3%, and clinical benefit, defined by stable disease, was observed in only 13% of the 68 patients enrolled [87].

### 6.2. Agents Targeting NFκB Signalling

Abnormal NFκB activation in DLBCL is often the result of oncogenic events affecting the upstream signaling network. Constitutive activation of NFκB is a hallmark of ABC-DLBCL. Lenalidomide alters the NFκB pathway by targeting Cereblon, a component of the E3 ubiquitin ligase multiprotein complex. It is an oral immunomodulatory imide anti-neoplastic agent that downregulates IRF4 protein downstream of Cereblon, the drug’s target. Multiple studies have shown lenalidomide to be effective in DLBCL, especially the ABC subtype [88,89,90].

### 6.3. Agents Targeting PI3K-AKT-mTOR Pathway

Abnormalities in the phosphoinositide 3-kinase (PI3K)/AKT/mammalian target of rapamycin (mTOR) signalling cascade, which plays a critical role in regulating cell proliferation, metabolism and survival, have been identified in DLBCL.

PI3K belongs to a family of lipid kinases, which are enzymes that generate signalling molecules by phosphorylating phosphatidylinositol lipids. The activation of PI3K-dependent pathways represents a crucial event of BCR/pre-BCR signaling. Class 1 PI3K is responsible for phosphorylating phosphatidylinositol-4.5-biphosphate (PIP2) to phosphatidylinositol (3,4,5)-triphosphate (PIP3). PTEN counteracts PI3K function by dephosphorylating PIP3 back to PIP2. Mutations or deletions resulting in the loss of PTEN activity are present in DLBCL. Class 1 PI3Ks are heterodimeric molecules composed of a p110 catalytic subunit and a shorter regulatory subunit (p85). The Class 1A p110 catalytic unit has 3 variants (p110α, p110β, p110δ). Class 1B PI3K is comprised of a p101 regulatory unit and a p110γ catalytic subunit.

The first PI3Kδ inhibitor, idelalisib, had disappointing outcomes in DLBCL clinical trials [91]. Duvelisib is a novel dual inhibitor of PI3Kδγ that has shown limited activity in aggressive NHLs compared with indolent NHLs [92]. Copanlisib is a pan-class 1 PI3K inhibitor, with the highest selectivity for the PI3Kα and PI3Kδ isoforms. A phase II study using copanlisib monotherapy demonstrated an ORR of 19.4% among the 67 DLBCL patients enrolled [93]. Clinical trials investigating the use of copanlisib in combination with R-CHOP, bendamustine, venetoclax, and nivolumab are ongoing.

mTOR inhibitors are an active area of investigation in DLBCL and have demonstrated modest responses in relapsed/refractory patients. A phase II trial of the oral mTOR inhibitor, everolimus showed a 30% overall response rate in the 47 DLBCL patients who were enrolled [94].

AKT inhibitors, such as capivasertib, are being investigated in DLBCL and show promise in combination therapies, especially with agents like the BCL-2 inhibitor venetoclax [95].

### 6.4. Agents Targeting BCL2 Signaling

Venetoclax, a BCL2 inhibitor, is primarily used in combination regimens in patients with DLBCL. The phase 2 CAVALLI study assessed the efficacy and safety of venetoclax with R-CHOP in untreated DLBCL and included a subgroup of patients who demonstrated BCL2 overexpression by immunohistochemistry. The complete response rate at the end of treatment was 69% in the overall population and was maintained in the BCL2+ IHC subgroup [96]. Preliminary results from the BO42203 trial, which evaluated the use of venetoclax with polatuzumab vedotin (an antibody–drug conjugate that targets CD79b) and R-CHP, have shown high rates of complete response in untreated high-risk BCL2-positive DLBCL patients [97]. Trials investigating the use of venetoclax in combination with other therapies for R/R DLBCL are also ongoing.

### 6.5. Agents Targeting Epigenetic Pathways

Epigenetic regulators, including histone and DNA modifiers, play a key role in the pathogenesis and treatment resistance of DLBCL. Novel agents directed against these pathways are currently in development and clinical evaluation, providing promising therapeutic opportunities for patients with R/R DLBCL. Tazemetostat is a selective and reversible small-molecule EZH2 inhibitor approved for R/R follicular lymphoma and has been investigated in DLBCL. Combination therapies with tazemetostat are being studied to improve efficacy. Tazemetostat in combination with R-CHOP was found to be safe and well tolerated in a phase 2 clinical trial of DLBCL patients aged 60–80 years. The complete metabolic response rate in this study was 75.4% [98].

Histone deacetylase (HDAC) inhibitors are being investigated as potential therapies for DLBCL, with early studies showing preclinical and clinical activity, particularly in combination therapies. HDAC inhibitors work by influencing gene expression and leading to cell-cycle arrest and apoptosis. A phase 2 study performed in China showed that chidamide plus rituximab was clinically effective, with an acceptable toxicity profile in elderly patients aged over 65 years with R/R DLBCL [99]. The overall response rate was 40% in the DLBCL group.

DNA methyltransferase (DNMT) inhibitors block the activity of enzymes responsible for adding methyl groups to DNA. Inhibiting this process leads to DNA demethylation, thereby restoring the expression of genes that were previously silenced through aberrant methylation. Despite the theoretical rationale for using DNA methyltransferase inhibitors like decitabine in DLBCL, clinical studies have not demonstrated consistent hypomethylation effects [100].

### 6.6. Immune-Based Precision Therapies

Recent advances in molecular profiling have accelerated the transition from empiric immunochemotherapy to precision medicine in DLBCL, enabling treatment strategies that are increasingly tailored to the tumour’s genetic, immunological, and microenvironmental characteristics. Chimeric antigen receptor (CAR) T-cell therapy, bispecific antibodies, and antibody–drug conjugates have emerged as major therapeutic advances for patients with relapsed or refractory DLBCL, demonstrating durable clinical responses in molecularly selected populations. However, despite these promising developments, the application of these therapies in HIV-associated DLBCL remains largely extrapolated from studies conducted in HIV-unrelated populations.

Table 7 provides a summary of these emerging immune-based therapeutic strategies and illustrates how an improved understanding of DLBCL biology is being translated into precision medicine.

## 7. Treatment of DLBCL in HIV-Positive Patients

In HIV-positive patients, treatment strategies are shaped not only by lymphoma staging and histology but also by immunological status and ART adherence. In the pre-ART era, standard-dose chemotherapy carried a risk of fatal opportunistic infections, prompting the exploration of low-dose regimens with limited success [55,110]. The introduction of ART improved survival, allowing more aggressive treatment approaches. Studies have shown that the outcomes of HIV-positive DLBCL patients treated with CHOP approach those of HIV-negative patients with DLBCL [12]. Rituximab-CHOP remains a widely used first-line therapy, though the use of rituximab, which is standard in HIV-negative patients, requires caution in patients with CD4 counts below 50 cells/µL due to increased infection-related mortality.

The pathogenic landscape of HIV-associated DLBCL highlights a pattern of pathway convergence that identifies rational targets for precision therapeutics and supports the incorporation of pathway-directed therapies alongside standard immunochemotherapy in future treatment strategies. Constitutive NF-κB and BCR signalling are common pathogenic mechanisms in HIV associated DLBCL, underscoring the therapeutic relevance of BTK, SYK and PI3K inhibitors in this setting. The HIV matrix protein p17 further enhances PI3K/AKT/mTOR activation, providing a functional basis for pathway dependence even in the absence of genetic mutations thus supporting the investigation of PI3K and mTOR inhibitors in HIV-DLBCL. EBV-driven lymphomagenesis, through LMP-mediated activation of JAK/STAT and NF-κB signalling, is particularly enriched in HIV-positive patients and aligns with growing evidence supporting immune checkpoint blockade and JAK inhibition.

The applicability and efficacy of immune-based therapies in HIV-associated DLBCL require particular consideration because these approaches depend, to varying degrees, on an intact and functional immune effector compartment. PLWH exhibit persistent immune dysfunction including T-cell exhaustion, which may influence the manufacturing, expansion and long-term persistence of autologous CAR T-cell products. Current data on the effects of CAR T-cell therapy in HIV-associated DLBCL are extrapolated from case reports [111]. Clinical experience with CAR T-cell therapy in PLWH suggests that HIV infection itself does not necessarily preclude effective cellular immunotherapy, although infectious complications, the effects of prior therapy, and immune dysfunction require careful consideration. The efficacy of immune checkpoint inhibitors may be reduced in patients with low nadir CD4+ T-cell counts, as significant CD4+ T-cell depletion may impair the development of sustained anti-tumour immune response despite checkpoint blockade. This may be further influenced by the TME in HIV-associated DLBCL. Pre-existing immune activation and dysfunction also influence the risk of immune-related toxicities such as CRS and ICANS. Furthermore, lymphodepleting conditioning regimens used with some cellular therapies may increase the risk of opportunistic infections and therefore require close monitoring. HIV-related immune dysfunction creates a biological rationale for potentially altered efficacy, but current evidence is insufficient to determine whether efficacy is inferior, equivalent, or dependent on the degree of immune reconstitution.

Table 8 summarises the potential therapeutic vulnerabilities together with the current level of evidence for HIV-associated DLBCL.

## 8. Conclusions

DLBCL is a biologically heterogeneous disease in which advances in molecular profiling have transformed disease classification and accelerated the development of precision medicine. Although HIV-associated and HIV-unrelated DLBCL share many oncogenic pathways, accumulating evidence indicates that HIV-associated DLBCL evolves within a distinct biological context shaped by chronic immune activation, viral co-infections, immune dysregulation, and a unique TME. These differences may influence tumour evolution, therapeutic vulnerabilities, and treatment responses, highlighting the need for HIV-specific molecular and clinical investigations.

This review highlights important gaps in our understanding of HIV-associated DLBCL and underscores the limitations of extrapolating precision medicine strategies from HIV-negative populations to PLWH. To advance the therapeutic paradigm, future research must move beyond retrospective observational studies toward prospective, biologically stratified clinical trials that integrate genomic, epigenomic, and transcriptomic profiling with viral status, immune biomarkers, and TME characteristics. Equally important is the inclusion of PLWH in multicentre translational studies and therapeutic trials to validate HIV-specific biomarkers, evaluate novel targeted approaches and establish the long-term safety and efficacy of emerging therapies. Such efforts will be essential to refine molecular classification, identify clinically actionable therapeutic targets, and realise equitable precision medicine for patients with HIV-associated DLBCL.

## Figures and Tables

**Table 1:** Next-generation sequencing (NGS) molecular subtypes in DLBCL and their alignment with traditional cell-of-origin (COO) classifications [14,15,16,17].

NGS Molecular Subtype	Primary Genotype and Key Driver Mutations	Overlapping Traditional COO Category	Clinical Phenotype and Outcome Impact
**MCD**	*MYD88^L265P^*, *CD79B*	ABC (Highly enriched)	Aggressive clinical course; frequent extranodal or central nervous system (CNS) localization.
**BN2**	*BCL6* fusions, *NOTCH2*, *SPEN*, *CCND3*	Unclassified/ABC	Favourable, shares clear pathogenetic pathways with marginal zone lymphoma
**N1**	*NOTCH1* mutations	ABC (Exclusively enriched)	Poor prognosis; clinical behaviour parallels transformations observed in chronic lymphocytic leukaemia.
**EZB**	*BCL2* fusions/translocations, *EZH2*, *KMT2D*, *CREBBP*	GCB (Highly enriched)	Variable clinical outcomes; can be further stratified by *MYC* structural rearrangement status into high-risk groups.
**ST2**	*SGK1*, *TET2*, JAK2	GCB (Predominantly)	Highly favourable prognosis; demonstrates shared genomic features with T-cell histiocyte-rich large B-cell lymphoma.
**A53/TP53Mut**	*TP53* mutations/deletions, *B2M* mutations, *TP53BP1*	Spans ABC, GCB, and Unclassified	Uniformly dismal therapeutic response; genomic instability completely overrides any underlying “favourable” GCB lineage.

**Table 2:** Genetic lesions associated with GCB DLBCL [27,28,29,30,31,32,33,34,35].

Lesion	Proportion Affected	Mechanism of Action
**Chromosomal translocation involving BCL2-most common t (14;18)**	~30–35%	BCL2 is a critical anti-apoptotic protein that is typically downregulated in GCs. Chromosomal translocations disrupt this regulation by placing Ig enhancer elements adjacent to the BCL2 gene. This rearrangement overrides BCL6-mediated suppression and leads to constitutive BCL2 expression.
**Chromosomal translocations involving MYC [most common t (8;14)]**	~15%	MYC functions by controlling proliferation, cell growth, DNA replication and apoptosis. Translocations involving MYC gene brings the gene under the transcriptional control of the Ig enhancer.
**Amplification of MIGH1 region containing microRNA 17–92 cluster on chromosome 13q**	~12%	MicroRNA polycistron acts as an oncogene, enhancing oncogenesis by increasing survival and proliferation by inhibiting tumour suppressor Phosphatase and Tensin Homolog (PTEN) and proapoptotic protein BCL-2 like 11 (BIM).
**Mutations of Enhancer of Zeste Homolog 2 (EZH2) methyltransferase**	~22%	EZH2 is a component of the polycomb repressive complex 2. It is required for the formation of the GC and involved in controlling multiple genes (IRF 4, B lymphocyte maturation protein 1 (BLIMP1), Cyclin dependent kinase 1 (CDKN1)). Mutations result in enhanced ability of EZH2 to trimethylated histone H3.
**Deletions of PTEN on chromosome 10q**	~11%	PTEN is a potent tumour suppressor protein that counteracts PI3K function by dephosphorylating PIP3 back to PIP2.
**Mutations in Phosphoinositide 3-kinase (** **PI3K** **)**	~8%	Results in the loss of PTEN.
**Mutations in Tumour Necrosis Factor Receptor Superfamily Member 14 (TNFRSF14)**	~30%	TNFRSF14 belongs to the tumour necrosis factor receptor superfamily and plays a key role in immune regulation. Loss or silencing of TNFRSF14 disrupts its inhibitory signalling, resulting in autonomous B-cell activation and proliferation.

**Table 3:** Genetic Lesions associated with ABC DLBCL [36,37,38,39,40,41].

Lesion	Proportion Affected	Mechanism of Action
**Mutations in BCR signalling**	~20% (gain of function mutations in CD79b, CD79a) ~9% (mutations involving Caspase Recruitment Domain Family Member 11 (CARD11))	ABC-DLBCL displays “chronic active” BCR signalling. Leads to constitutive activation of NFκB pathway.
**Mutations of Myeloid Differentiation Primary Response 88** (**MYD88**)	~30%	Mutations of MYD88 leads to L265P mutant protein which promotes cell survival by activating NFκB signalling and JAK/STAT3 signalling cascade.
**Mutations of Tumour necrosis factor, alpha-induced protein 3** (**TNFAIP3**)	~30%	TNFAIP3 gene encodes for the negative NFκB regulator A20. Mutations in this gene contributes to lymphomagenesis by constitutively activating NFκB.
**Inactivating mutations and deletions of PRDM1 gene on chromosome 6q21**	~30%	PRDM1 gene encodes BLIMP1, a zinc finger transcriptional repressor. BLIMP1 suppresses GC master genes such as PAX5 and BCL6.

**Table 4:** Genetic Lesions associated with both GCB and ABC DLBCL [42,43,44,45,46,47,48].

Lesion	Proportion Affected	Mechanism of Action
**Epigenetic alterations:** **1. KMT2D (MLL) gene somatic mutations**	~30%	Somatic mutations result in inactivation of KMT2D/MLL methyltransferase.
**2. CREBBP/EP300 somatic mutations and/or deletions**	~39% GCB-DLBCL ~17% ABC DLBCL	Results in inactivation of acetyltransferase genes CREBBP and EP300. CREBBP and EP300 acetyltransferases act as transcriptional coactivators in multiple signalling pathways. Mutations result in the loss of their ability to acetylate BCL6 and p53.
**Deregulation of BCL6**	~66% (chromosomal translocations)	BCL6 is a transcriptional repressor belonging to the BTB/zinc finger family of transcriptional factors.
		Chromosomal translocations induce constitutive BCL6 expression
	~75% (mutations of 5^1^ regulatory region)	Mutations affecting the 5^1^ regulatory region impairs the negative autoregulatory loop through which BCL6 controls its own expression.
	~9% (MEF2B mutations)	MEF2B mutations enhance its transactivator function and hence BCL6 expression.
	~5% FBXO11 mutations/deletions	FBXO11 is a ubiquitin adaptor protein that normally targets BCL6 for proteasomal degradation. Loss of function mutations and/or deletions of FBXO11 results in decrease catabolism of BCL6.
**Mutations in Forkhead box 01 (FOXO1)**	8–10%	FOXO1 transcription factor is a key player in B-cell differentiation. Its activity is negatively regulated by PI3K-AKT and mTOR cascade. Mutations prevent inactivation of FOXO1 in response to PI3K signalling.

**Table 5:** Principal genomic, transcriptomic and microenvironmental differences between HIV-associated and HIV-unrelated DLBCL [29,66,69,73,74,75,76,77].

Category	HIV-Unrelated DLBCL	HIV-Associated DLBCL	Biological/Clinical Consequence
**Prognostic Subtyping**	Relies on Cell-of -Origin (COO) (GCB vs. ABC) to predict survival	Prognostic utility of COO appears reduced and requires further validation; conventional risk stratification fails.	Requires new, HIV-specific genomic biomarkers for risk assessment
**Mutational drivers**	Driven by Aberrant Somatic Hypermutation (ASHM)	ASHM appears less dominant, instead shows high microsatellite instability (MSI).	Points to distinct mechanisms of initial DNA damage and lymphomagenesis
**Viral oncogenesis**	EBV positivity is rare, (<5% in young adults)	EBV positivity is frequent (60–70%)- splitting the disease in 2 distinct genetic pathways. EBV + HIV+ cases driven primarily by oncogenes that activate host JAK/STAT and NFkB signalling networks. Frequent somatic mutations occur in the STAT3 and DDX3X. EBV-HIV+ cases driven by severe host genetic dysregulation, presenting mutational rates in epigenetic modifiers (KMT2D and TET2) and the PI3K/mTOR pathways.	EBV+ cases bypasses host mutations by using viral oncoproteins to activate JAK/STAT.
**Transcriptomic** **Program**	Variable metabolic signatures based on genetic subtype.	Emerging transcriptomic studies suggest enrichment of oxidative phosphorylation (OXPHOS), hypoxia and mTORC pathways.	Suggests potential for metabolic -targeted therapies. (e.g., mTOR inhibitors)
**Epigenetic Landscape**	Standard, subtype dependent histone modification profiles.	Marked DNA hypomethylation at specific gene promoters (e.g., HDAC9, FOXP2).	Highlights a heavily altered chromatin state unique to the HIV environment.
**Immune Evasion**	Intact or variable antigen presentation.	Severe loss of MHC-1 expression on malignant B cells.	Allows tumours to remain entirely invisible to host’s surviving T cells.
**Tumour Microenvironment** (**TME**)	Standard background inflammatory and immune cell infiltrations.	Contains highly specific reservoirs of naïve and atypical memory B cells creating a pro-survival niche.	The surrounding tissue actively promotes tumour progression, rather than fighting it.

**Table 6:** Comparative clinicopathological, molecular and therapeutic features of HIV-associated and HIV-unrelated DLBCL.

Feature	HIV-Unrelated DLBCL	HIV-Associated DLBCL	Precision Medicine Implications
**Underlying** **pathogenesis**	Predominantly tumour-intrinsic genetic and epigenetic alterations acquired during germinal centre maturation.	Chronic immune activation, persistent antigenic stimulation, HIV-mediated immune dysregulation and frequent viral co-infections.	Different mechanisms of lymphomagenesis may require different biomarker driven therapeutic strategies.
**Cell-of-origin** (**COO**)	Both GCB and ABC subtypes.	Predominantly ABC, although GCB tumours do occur.	COO classification remains important for molecular stratification and targeted therapy selection.
**EBV association**	Uncommon.	Common, contributes to immune escape and oncogenic signalling.	Supports investigation of immune checkpoint inhibitors, EBV-directed immunotherapy and adoptive T-cell therapies.
**Dominant molecular drivers**	MYD88, CD79B, EZH2, BCL2, CREBBP, EP300, TP53 and other recurrent driver mutations.	MYC, BCL6, TP53, PIM1, PAX5, EBV-positive tumours frequently show STAT3, DDX3X and NK-kB/JAK-STAT pathway activation.	Molecular profiling may identify pathway-specific therapeutic vulnerabilities.
**Aberrant signalling pathways**	BCR/NF-kB, PI3K/AKT/mTOR, BCL2, epigenetic and apoptotic pathways.	Chronic BCR/NF-kB activation, PI3K/AKT/mTOR activation, JAK/STAT activation, immune checkpoint dysregulation.	Provides rationale for BTK, PI3K, mTOR, BCL2 and JAK inhibitors, depending on the molecular context.
**Tumour microenvironment**	Variable immune microenvironment with less pronounced systemic immune dysfunction.	Chronic inflammation, T-cell exhaustion, macrophage activation, impaired immune surveillance and cytokine dysregulation.	Immune profiling may help identify patients likely to benefit from immunotherapy.
**Genomic landscape**	Well characterized genomic subtypes (e.g., MCD, BN2, EZB, ST2, N1, A53).	Greater genomic complexity in some studies, EBV-dependent molecular heterogeneity, limited genomic datasets due to exclusion from clinical trials.	Expanded genomic studies are required to define HIV-specific molecular subtypes.
**Clinical presentation**	More heterogenous presentation depending on molecular subtype.	Younger age, advanced stage, extranodal disease, higher tumour burden and immunodeficiency.	Risk-adapted treatment should integrate HIV status, immune function and tumour biology.
**Standard therapy**	R- CHOP (rituximab, cyclophosphamide, doxorubicin, vincristine, prednisone) remains first line treatment.	R-CHOP plus antiretroviral therapy remains standard, with careful consideration of CD4 count, infection risk and drug interactions.	Optimisation of therapy requires balancing lymphoma control with immune preservation.
**Emerging precision therapies**	Increasing evidence supports molecularly selected targeted therapies and immune based approaches.	Potential roles for BTK inhibitors, PI3K inhibitors, JAK inhibitors, immune checkpoint inhibitors, CAR T-cell therapy, bispecific antibodies and antibody-drug conjugates; HIV-specific evidence remains limited.	Prospective HIV-inclusive trials are needed to validate precision medicine strategies.
**Current challenges**	Tumour heterogeneity and therapeutic resistance.	Underrepresentation in genomic studies and clinical trials; limited biomarker validation; interactions with antiretroviral therapy.	Inclusion of PLWH in molecular studies and clinical trials is essential to achieve equitable precision oncology.

**Table 7:** Emerging immune-based therapeutic strategies for DLBCL [101,102,103,104,105,106,107,108,109].

Therapeutic Strategy	Biological Rationale	Representative Agents	Current Limitations
**Anti-CD19 CAR T-cell therapy**	CD19-directed elimination of malignant B cells; overcomes chemotherapy resistance.	Axicabtagene ciloleucel, Tisagenlecleucel	Cytokine release syndrome (CRS), immune effector cell-associated neurotoxicity syndrome (ICANS), specialised manufacturing, high cost.
**Bispecific Antibodies**	Redirect endogenous T cells toward CD20-positive lymphoma cells independent of antigen presentation.	Mosunetuzumab, Epcoritamab, Glofitamab	CRS, neutropenia, infections, HIV-specific clinical data remain limited.
**Bispecific T-cell engager (BiTE)**	Simultaneous engagement of CD19 and CD3 to activate cytotoxic T cells.	Blinatumomab	Continuous infusion, short half-life, CRS, ICANS
**Antibody–drug conjugates (ADCs)**	Targeted delivery of cytotoxic agents to malignant B cells while limiting systemic toxicity.	Polatuzumab vedotin (CD79b)	Peripheral neuropathy, cytopenias, gastrointestinal toxicity
**Immune checkpoint inhibitors**	Restore anti-tumour immunity by blocking inhibitory immune checkpoint pathways (PD-1/PD-L1), thereby reversing T-cell exhaustion and enhancing cytotoxic T-cell activity. Particularly relevant in EBV-positive tumours with increased PD-L1 expression and immune escape.	Pembrolizumab, Nivolumab, Atezolizumab, Durvalumab	Modest efficacy in unselected DLBCL, immune-related adverse events, lack of predictive biomarkers, and underrepresentation of people living with HIV in clinical trials.

**Table 8:** Precision medicine opportunities arising from the unique biology of HIV-associated DLBCL [83,84,92,93,94,98,99,106,107,108,109,110,111,112,113,114,115,116].

Biological Feature	Mechanistic Rationale	Potential Precision Medicine Strategy	Current Evidence
**Chronic Immune Activation**	Persistent antigenic stimulation promotes AID overexpression, B cell stimulation and genomic instability.	Immunomodulatory agents combined with standard chemotherapy.	Early phase clinical trials show feasibility, HIV-specific evidence remains limited.
**Epstein-Barr Virus (EBV) Co-infection**	Viral proteins (LMP1, EBNA) activate survival pathways, immune evasion and PD-L1 expression.	EBV-specific cytotoxic T-lymphocytes (CTLs), adoptive cell strategies, or EBV-targeted prophylactic/therapeutic vaccines.	Early-phase studies demonstrate promise in EBV-associated lymphoproliferative disorders.
**Immune Checkpoint Upregulation (PD-1/PD-L1)**	HIV and EBV promote T-cell exhaustion and immune escape through PD-1/PD-L1 signalling.	PD-1/PD-L1 checkpoint inhibitors, alone or in combination with chemotherapy.	Phase II studies show activity, particularly in EBV-positive disease, HIV specific data remain limited.
**Aberrant B-cell Receptor (BCR) Signalling**	Mutations involving MYD88, CD79B and CARD11 promote constitutive survival signalling.	BTK inhibitors and other BCR pathway inhibitors.	Protocol adaptations inserting daily ibrutinib alongside DA-EPOCH backbones. Most current trial populations remain centred on HIV-negative cohorts.
**PI3K/AKT/mTOR Pathway Activation**	HIV infection and chronic inflammation activate PI3K/mTOR pathway.	PI3K inhibitors and mTOR inhibitors	Preclinical and basic translational models are encouraging; prospective HIV-specific clinical phase protocols are largely unexecuted.
**Epigenetic Dysregulation**	Altered histone modification and DNA methylation contribute to lymphomagenesis.	EZH2 inhibitors and HDAC inhibitors.	Clinical benefit demonstrated in selected HIV negative subtypes. Utility in HIV-associated cohorts remain unmapped.
**CD19 expression**	Preserved CD19 expression enables targeted cellular therapy.	Autologous anti-CD19 CAR T-cell therapy products (e.g., axicabtagene ciloleucel) or CD19-directed antibody-drug conjugates (ADCs).	Prospective multicentre cohort data and post-approval tracking demonstrate high feasibility, safety and comparable complete response (CR) rates in PLWH with HIV viral load is suppressed.
**CD20 expression**	Persistent CD20 expression supports antibody-mediated cytotoxicity.	Next-generations CD20-targeted monoclonal antibodies and CD20xCD3 bispecific T-cell engagers	Phase I/II clinical evaluation of bispecific constructs shows high efficacy in relapsed/refractory settings; however, data specifically focused on HIV-positive patients is highly limited.
**Tumour microenvironment remodelling**	Immune dysregulation and macrophage polarization promote tumour progression and immune escape.	Combination immunotherapy, macrophage checkpoint targets (e.g., anti-CD47) and cytokine targeted approaches.	Predominantly preclinical and early translational evidence.

## Data Availability

No new data were created or analyzed in this study. Data sharing is not applicable to this article as it is a review of existing literature.

## Abbreviations

The following abbreviations are used in this manuscript:

DLBCL	Diffuse Large B-cell Lymphoma
NHL	Non-Hodgkin Lymphoma
HIV	Human Immunodeficiency Virus
ART	Antiretroviral therapy
PLWH	People living with HIV
GC	Germinal centre
BCL2	B-cell lymphoma 2
SHM	Somatic hypermutation
BCR	B-cell receptor
AID	Activation-induced cytidine deaminase
BCL6	B-cell lymphoma 6
MYC	Myelocytomatosis oncogene
GEP	Gene expression profiling
GCB	Germinal centre B-cell
ABC	Activated B-cell
PAX5	Paired Box Gene 5
EBV	Epstein–Barr virus
TME	Tumour microenvironment
BTK	Bruton tyrosine kinase
SYK	Spleen tyrosine kinase
FFP	Freedom from progression
P13K	Phosphoinositide 3-kinas
